# The Extracellular NADome Modulates Immune Responses

**DOI:** 10.3389/fimmu.2021.704779

**Published:** 2021-08-04

**Authors:** Valentina Audrito, Vincenzo Gianluca Messana, Lorenzo Brandimarte, Silvia Deaglio

**Affiliations:** Laboratory of Cancer Immunogenetics, Department of Medical Sciences, University of Turin, Turin, Italy

**Keywords:** nucleotides, NAD, signaling, DAMPs, immune cell regulation, immunometabolism, tumor microenvironment

## Abstract

The term NADome refers to the intricate network of intracellular and extracellular enzymes that regulate the synthesis or degradation of nicotinamide adenine dinucleotide (NAD) and to the receptors that engage it. Traditionally, NAD was linked to intracellular energy production through shuffling electrons between oxidized and reduced forms. However, recent data indicate that NAD, along with its biosynthetic and degrading enzymes, has a life outside of cells, possibly linked to immuno-modulating non-enzymatic activities. Extracellular NAD can engage puriginergic receptors triggering an inflammatory response, similar - to a certain extent – to what described for adenosine triphosphate (ATP). Likewise, NAD biosynthetic and degrading enzymes have been amply reported in the extracellular space, where they possess both enzymatic and non-enzymatic functions. Modulation of these enzymes has been described in several acute and chronic conditions, including obesity, cancer, inflammatory bowel diseases and sepsis. In this review, the role of the extracellular NADome will be discussed, focusing on its proposed role in immunomodulation, together with the different strategies for its targeting and their potential therapeutic impact.

## Introduction: The Many Faces of NAD, From Energetic Factor to Danger Signal

NAD is an essential intracellular metabolite with key roles in energy metabolism and electron transfer (1–7). In addition, NAD is a cofactor for different families of enzymes, including sirtuins and poly-ADP-ribosyl polymerases (PARPs). NAD can be present outside of cells, with levels fluctuating widely in response to extracellular signals (8–11). A firm observation is that under steady state extracellular (e)NAD levels are thousands of times lower (nM) compared to the intracellular ones (µM-mM) (7, 12–16).

However, during conditions of cellular stress, such as those observed in an inflamed microenvironment, or during hypoxia, or in conditions of shear stress due to physical distortion, plasma membrane damage, stress elicited by cytotoxic agents, NAD concentrations may rapidly spike. This observation, together with the finding that some purinergic receptors are activated by NAD suggested that eNAD serves as a “danger signal” that alerts the immune system to tissue damage (8–10, 12, 17–20). According to this view, eNAD could be considered as damage-associated molecular pattern molecule (DAMP), able to activate the innate immune system, like what has been shown for pathogen-associated molecular patterns (PAMPs) (18, 21–24). For example, released eNAD from active neuronal cells can serve as neurotransmitter and neuromodulator (25–27); or in a mouse model of inflammation, induced by injection of polyacrylamide beads, eNAD reached a concentration of 10mM acting as danger signal (28).

NAD release may occur by several mechanisms involving active exocytosis, or diffusion through transmembrane transporters (e.g., pannexin, connexin) in living cells, or passive leakage across the membrane from necrotic or injured cells (15, 29–32).

Homeostasis is rapidly restored through a scavenging circuit operated by nucleotide-catabolizing enzymes that produce the immunosuppressant adenosine (ADO) and inosine, which can re-enter the cell, reconstituting the nucleotide pool (5, 33–36). All these mechanisms of nucleotide/nucleoside release to alert or switch off the immune system, respectively, are enhanced during acute and chronic inflammation, including cancer (29, 37, 38). Even though very unlikely, eNAD synthesis has not been conclusively ruled out, also in consideration of the presence of several key NAD biosynthetic enzymes (NBEs) (16, 39).

## Intracellular and Extracellular NAD-Metabolizing Machinery

**Figure 1:**
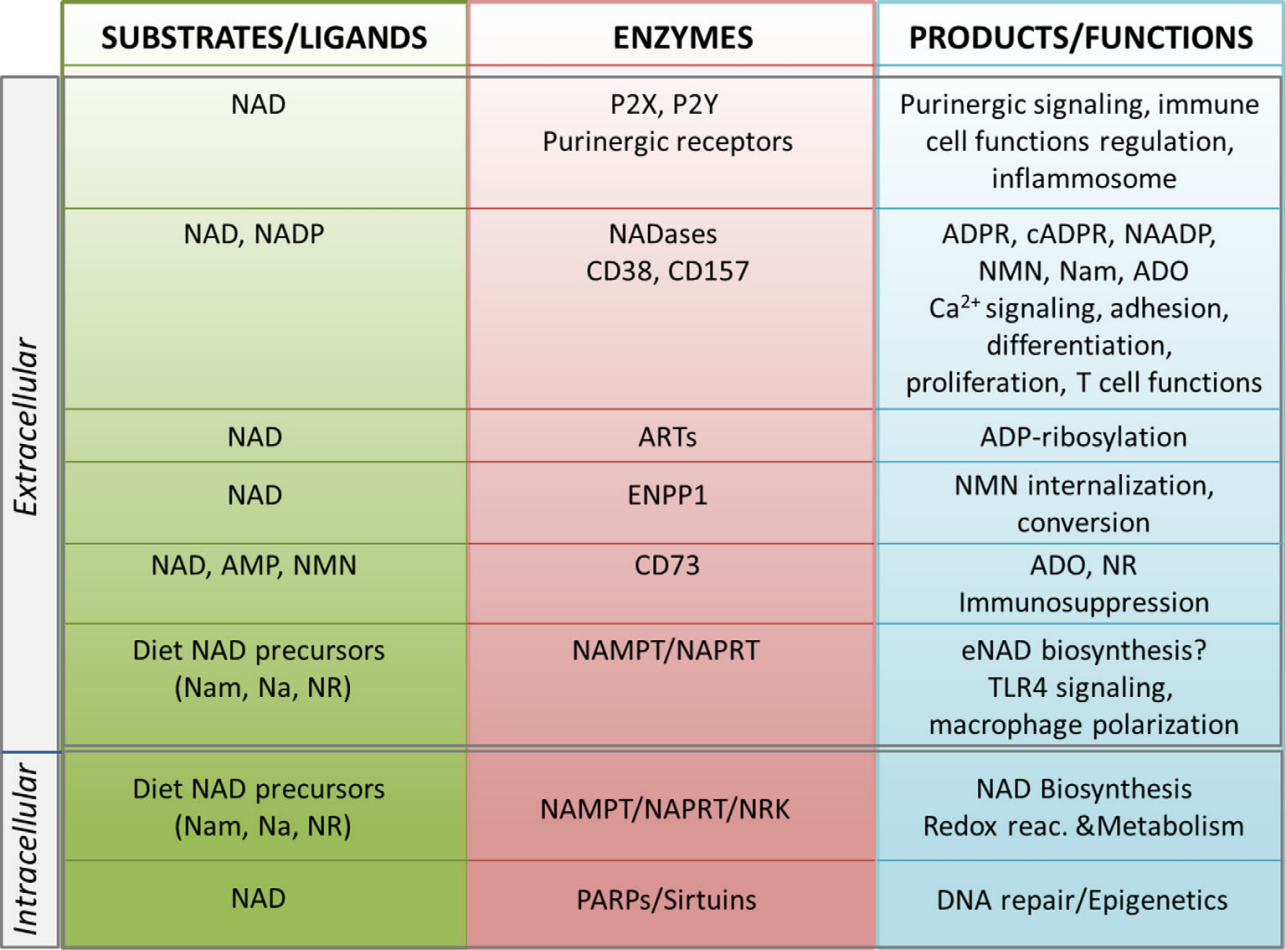
Schematic representation of the NADome. Schematic representation of the network of substrates/ligands, NAD-metabolizing cell surface and intracellular enzymes and their products in the extracellular and intracellular space. Biological functions regulated by NAD-related enzymes and products are listed. NAD, nicotinamide adenine dinucleotide; NADP, NAD phosphate; eNAD, extracellular NAD; Nam, nicotinamide; NR, nicotinamide riboside; Na, nicotinic acid; NAMPT, nicotinamide phosphoribosyltransferase; NAPRT, nicotinate phosphoribosyltransferase; NRK, nicotinamide riboside kinase; ARTs, mono adenosine diphosphate (ADP)-ribose transferases; PARPs, poly ADP-ribose polymerases; ADPR, ADP ribose; cADPR, cyclic ADP ribose; NAADP, nicotinic acid adenine dinucleotide phosphate; Ca^2+^, calcium; NMN, nicotinamide mononucleotide; ADO, adenosine; AMP, adenosine monophosphate; ENPP1; ectonucleotide pyrophosphatase/phosphodiesterases; TLR4, toll-like receptor 4.

The biosynthesis of NAD takes place in different locations in the cell, through one *de novo* pathway starting from the catabolism of tryptophan, and *via* degradation of vitamin B3 precursors. The latter is considered a salvage pathway that occurs through the metabolism of three precursors [i.e. nicotinic acid (Na), nicotinamide (Nam) and nicotinamide riboside (NR)]. In the majority of tissues, intracellular NAD is generated mostly from Nam, which is the degradation product of all NAD-consuming signaling reactions (6, 40–42). Under normal conditions >70% of the cellular NAD content is stored and is utilized in the mitochondria primarily for metabolic purposes (16, 43). The cytosolic and nuclear NAD pools serve primarily to sustain activity of PARPs and sirtuins, which are NAD-dependent enzymes with key roles in regulating DNA repair and epigenetic controlling of gene transcription, respectively (**Figure 1**) (7, 44, 45). NAD levels can therefore restrict the activity of these two classes of NAD-metabolizing enzymes. Intriguingly, NAD can rapidly shuttle between different cellular compartments to reconstitute the pool that allows enzyme activation, as has recently been shown (46). When in the extracellular space, eNAD functions are linked to the modulation of cell surface P2X and P2Y purinergic receptor families, thereby acting in an apparently enzyme-independent way and eliciting pro-inflammatory immune responses (**Figure 1**). In addition, within the extracellular space, a complete network of different ectonucleotidases can rapidly hydrolyze eNAD generating intermediates that modulate signaling, cell metabolism, adhesion, migration and activate immunoregulatory circuits (14, 39, 47), as summarized in **Figure 1**. eNAD is degraded by different classes of ectoenzymes: the NADases CD38 and CD157 (48–50), the ADP-ribosyltransferases (ARTs) (51), the Ectonucleotide Pyrophosphatase/Phosphodiesterase 1 (ENPP1) and the ecto-5′-nucleotidase CD73 (34, 52, 53). NADase, ENPP1 and CD73 can lead to the formation of ADO, a potent natural immunosuppressive factor mediating the activation of the inhibitory P1 purinergic receptors (34, 54, 55). In addition, eNAD can be cleaved to nicotinamide mononucleotide (NMN) and subsequently dephosphorylated to NR by CD38 and CD73 (53, 56, 57). All these intermediates can enter the cell as NAD precursors and can be used by NBEs, reconstituting the intracellular pool (57, 58) (**Figure 1**).

In the next sections of this review, we will summarize the role of eNAD, its derived-metabolites and a set of NAD-dependent enzymes, giving examples of their role in the regulation of specific immune responses.

## eNAD and Purinergic Receptors

The idea of purinergic signaling, i.e., of nucleotides acting as extracellular signaling molecules, was initially put forward by the seminal work of Geoff Burnstock in 1972 (59, 60).

**Figure 2:**
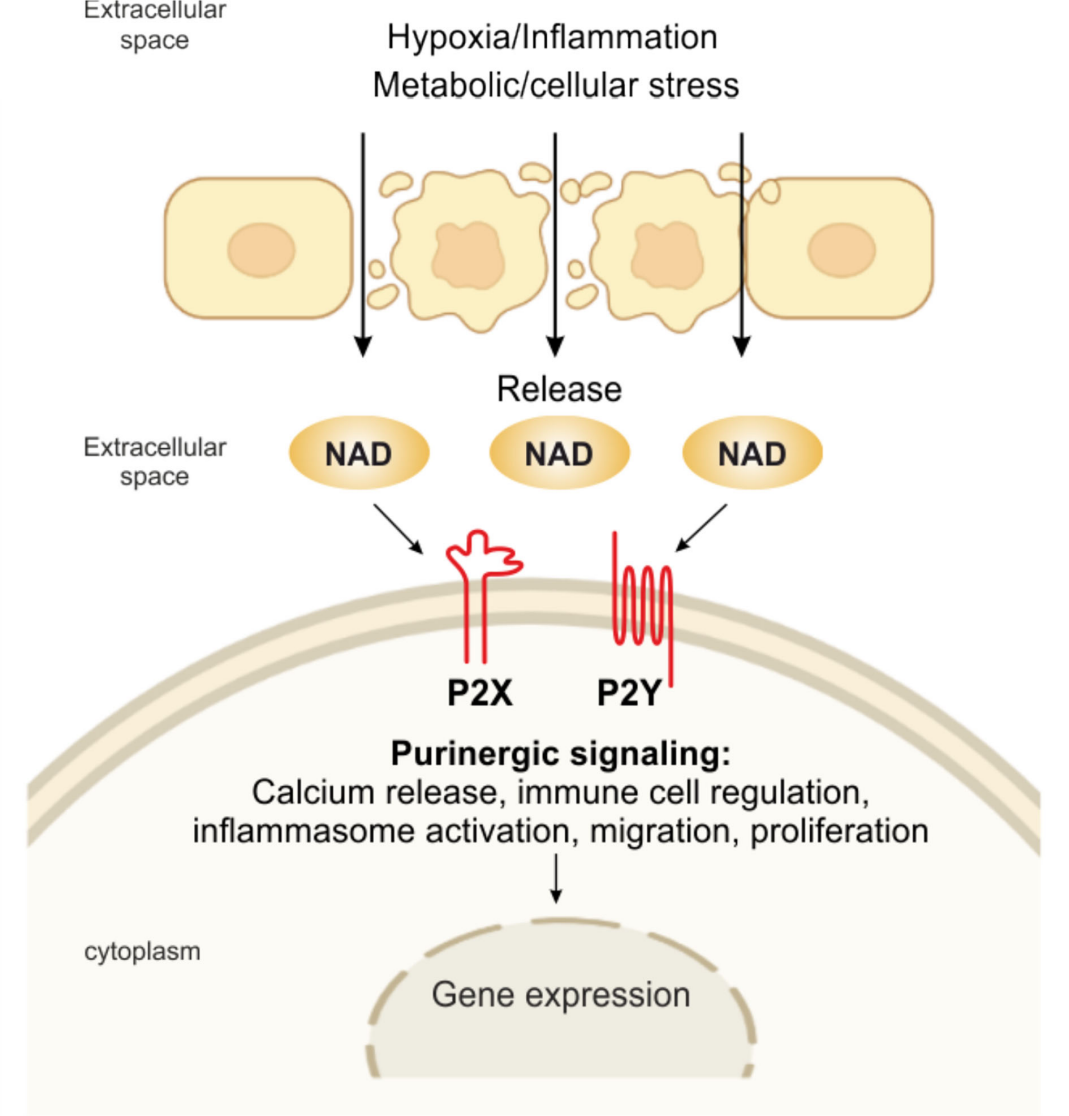
eNAD and purinergic signaling. Pathological or physiological stimuli, including hypoxic and inflammatory conditions, metabolic and cellular stress, promote the release of NAD from the cell. NAD can then bind to P2X (isoforms P2X1, P2X4, P2X7) and P2Y (isoforms P2Y1, P2Y11), activating several intracellular signaling and modulating immune responses.

Since then, this complex network of receptors has progressively been unveiled to reveal seven evolutionarily conserved subtypes of the P2X ion channel receptors and eight subtypes of the P2Y G protein-coupled receptor, all with roles in immune cell activation (5, 24, 61). On the contrary, four subtypes of the ADO P1 receptors on effector T cells have immunosuppressive effects. Shifting the balance from pro-inflammatory P2R signaling to anti-inflammatory P1R signaling or vice versa, the purinergic signaling system fine-tunes immune cell functions (5). eNAD can bind different subtypes of purinergic P2 receptors as summarized in **Figure 2**.

For example, eNAD activates human granulocytes by binding P2Y11 and triggering: (i) overproduction of cyclic (c)AMP, (ii) activation of protein kinase A, (iii) stimulation of ADP-ribosyl cyclase and overproduction of cyclic ADP-ribose (cADPR), a universal calcium (Ca^2+^) mobilizer, and (iv) influx of extracellular Ca^2+^, ultimately causing increased proliferation and migration (62). eNAD can bind P2Y1 and P2Y11 in human monocytes activated with lipopolysaccharide (LPS), triggering a transient rise in intracellular Ca^2+^, which is caused by a release of Ca^2+^ from IP (3)-responsive intracellular stores and an influx of extracellular Ca^2+^ (63). eNAD has also been identified as an agonist at P2Y1 receptors in human embryonic kidney (HEK) cells and mouse colonic muscle (27, 64). Moreover, binding to postsynaptic P2Y1 receptors, like ATP, eNAD also acts as a neurotransmitter, released by stimulated terminals of mammalian central nervous system and peripheral nervous system neurons (65). In addition, it has been shown that purinoceptors, including P2X1, P2X4, and P2X7, are engaged in eNAD-mediated signaling (27, 63, 66). However, more experimental data should be published to confirm this direct binding of NAD *per se.*

eNAD may also engage P2X7R receptors, the main eATP receptor, extensively studied in the context of inflammation and immunity (24). P2X7R signaling is a major regulator of the intensity and duration of inflammatory responses (24, 67, 68). The receptor/channel is prominently expressed on all cells of innate and adaptive immunity and aberrant signaling has been linked to diverse inflammatory and autoimmune diseases, as recently reviewed in (5, 24). P2X7R signaling mediates NLR family pyrin domain containing 3 (NLRP3) inflammasome activation, cytokine, and chemokine release [i.e., interleukin (IL)-1β, tumor necrosis factor (TNF), IL-6, monocyte chemoattractant protein-1 (MCP-1/CCL2)], T lymphocyte survival and differentiation, transcription factor activation, and cell death (24, 69, 70). At inflammatory sites, P2X7R could also be bound directly by alternative ligands, including eNAD that accumulates at sites of inflammation and tissue damage (28). In murine T lymphocytes, eNAD serves as an ADP-ribose donor to ADP-ribosylate the P2X7R at arginine 125, close to the ATP-binding pocket (71). This reaction, catalyzed by the plasma membrane enzyme ART2.2, causes long-lasting activation of mouse P2X7R, negatively affecting T-regulatory (Treg) and natural killer T (NKT) cell survival and arguing in favor of a direct role of eNAD in the pathophysiological mechanism of P2X7R activation. The reduction of Treg function *via* NAD-induced gating of P2X7 can be employed *in vivo* as a strategy to promote the antitumor response of effector T cells. Systemic injection of NAD results in the selective depletion of Tregs *via* NAD-mediated activation of P2X7, which enhances anticancer immune responses in several mouse tumor models (20, 72). While highly interesting and potentially relevant for human immune responses, this mechanism of P2X7R activation *via* eNAD is demonstrated only in mouse models: additional research is needed to determine whether it is relevant for human immune responses too.

## eNAD Degradation-Signaling System in Regulating Immune Responses

**Figure 3:**
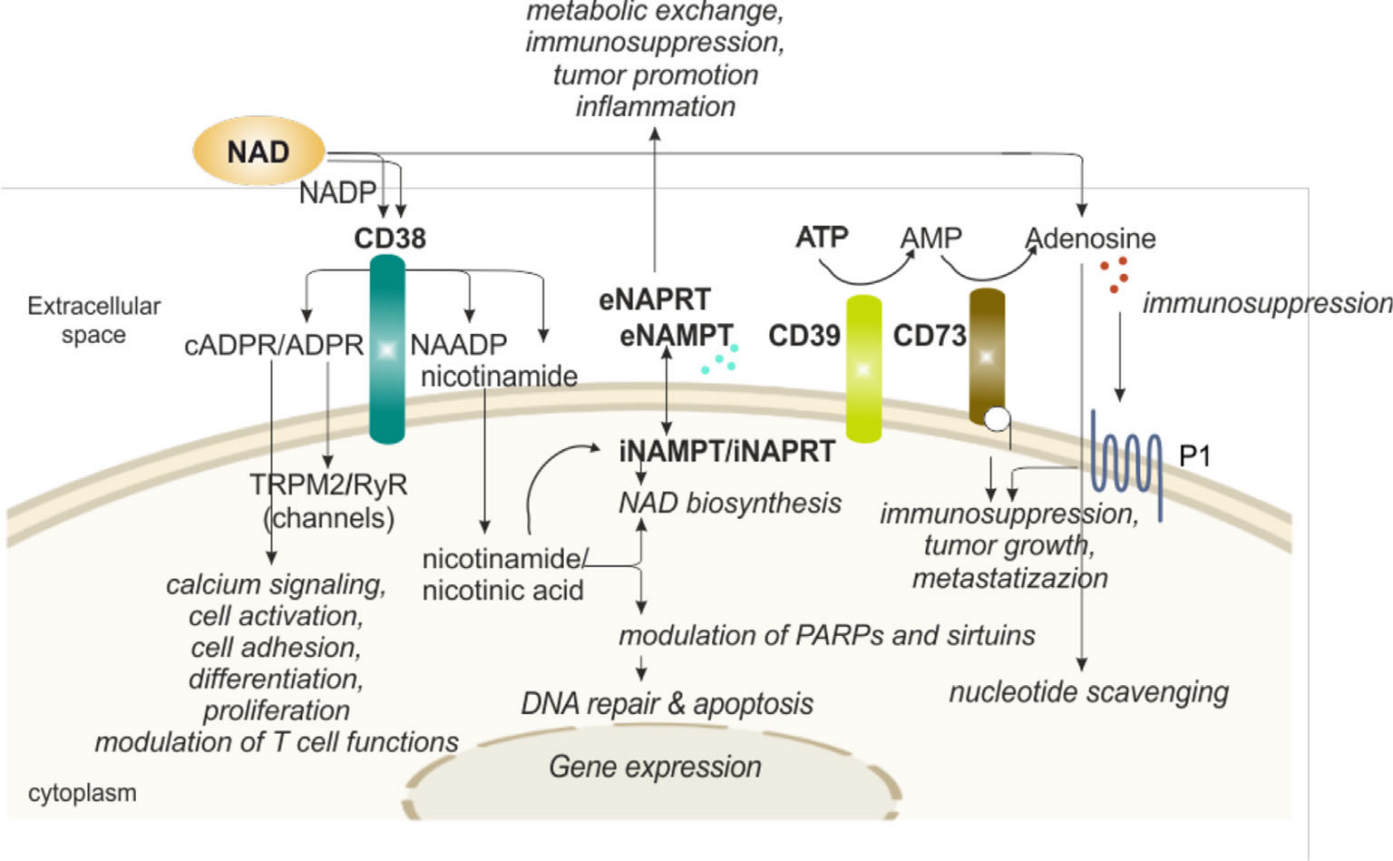
eNAD and enzymatic/functional extracellular machinery in regulating immune responses. Extracellular NAD can also be metabolized by a series of enzymes of the cell surface that are involved in scavenging of nucleotides. The end product of the reaction, adenosine, can then be internalized and reconverted to related nucleosides (e.g., ATP or NAD). In particular, CD38 hydrolyzes NAD to generate intermediates (cADPR and ADPR), potent intracellular Ca^2+^-mobilizing agents, through binding RyR or TRPM2 receptors. CD38 activation induces migration, proliferation, and modulation of immune responses, specifically T cell functions, as detailed in the text. In addition, CD38 activity releases nicotinamide (the main NAD precursor) that can be internalized into the cell and, together with a second precursor nicotinic acid, converted in NAD by NAMPT and NAPRT activities, respectively, increasing intracellular NAD levels and affecting sirtuins and PARPs (NAD-consuming enzymes) functions. NAMPT and NAPRT can be secreted/released in the extracellular space acting as cytokine-like proteins. Finally, NAD and ATP can be converted in adenosine. ATP is metabolized by CD39 to AMP that is further hydrolyzed by ecto-5’-nucleotidase/CD73 which promotes the formation of adenosine. Adenosine then activates adenosine receptors (purinergic receptor P1). The final outcome depends on the relative concentrations of substrates and products and on the expression of nucleotide-metabolizing ecto-enzymes and NAD-biosynthetic enzymes. NAD, nicotinamide adenine dinucleotide; NADP, NAD phosphate; NAMPT, nicotinamide phosphoribosyltransferase; NAPRT, nicotinate phosphoribosyltransferase; PARPs, poly ADP-ribose polymerases; ADPR, ADP ribose; cADPR, cyclic ADP ribose; NAADP, nicotinic acid adenine dinucleotide phosphate; ATP, adenosine triphosphate; AMP, adenosine monophosphate; P1, adenosine purinergic receptor; RYR, ryanodine receptors; TRPM2, transient receptor potential melastatin-related 2; i, intracellular; e, extracellular.

One of the reasons why eNAD levels are generally low is that there are several extracellular enzymes that rapidly transform it, guaranteeing recycling of a high energy molecule through the generation of products that can be easily up-taken by cells. The intermediates, however, have a life of their own as signaling molecules, thereby modulating activity of immune cells. The best-known NAD-degrading/signaling systems rely on the activity of CD38, an immunomodulatory enzyme (**Figure 3**).

Human CD38, the main member of the NADase/ADPR cyclase family that includes also CD157/*BST-1*, is a surface glycoprotein characterized by a relatively large extracellular domain that contains the catalytic site, a single transmembrane pass, and a short cytoplasmic tail (73, 74). CD38 is a multifunctional ectoenzyme, involved in the catabolism and degradation of eNAD (under normal pH) and NAD phosphate (NADP, under acidic pH), producing ADP ribose (ADPR) together with signaling metabolites involved in intracellular Ca^2+^ mobilization. The main catalytic activity is the NAD glycohydrolase that generates Nam and ADPR. CD38 can also act as NAD cyclase, producing cADPR, which is then hydrolyzed to ADPR. Lastly, in the presence of NADP and Na, under acidic pH levels, CD38 can generate nicotinic acid adenine dinucleotide phosphate (NAADP) (49). The finding of an extracellular enzymatic activity of CD38 leading to the generation of messengers that enter cells to induce intracellular Ca^2+^ fluxes remains an unsolved “topological paradox” (49, 75). More recent data have enriched the picture by showing that CD38 can also be found in the nucleus and mitochondrial membrane and that a soluble form of CD38 is most likely present in the cytoplasm, leading to the hypothesis of a compartmentalized generation of NAD-derived signaling metabolites (49, 76–78). ADPR, cADPR and NAADP share the ability to mobilize Ca^2+^ ions from intracellular stores: cADPR binds to ryanodine receptors (RyR) expressed on the endoplasmic reticulum, ADPR binds to membrane melastatin related transient receptor potential cation channels TRPM2 (49, 79) and NAADP binds to receptors expressed by acidic organelles, such as lysosomes, suggesting a role as Ca^2+^ messenger in the endocytic pathway (80). It is therefore likely that during an immune response, NAD, released outside of cells due to local conditions of inflammation and cellular damage is converted into Ca^2+^-active metabolites through the action of CD38 expressed by activated lymphocytes, which in turn contribute to lymphocyte activation through Ca^2+^ signaling (80–82).

There is a second alternative possibility that is gaining momentum in the context of tumor immunosuppression. According to this hypothesis, ADPR could also be short-circuited to ADO *via* the action of CD203, which generates AMP from ADPR and CD73 (53, 83, 84), which cleaves the last phosphate, generating ADO (49). In this way, CD38 could contribute to the generation of a tumor-favorable environment, as recently demonstrated in tumors characterized by a large T cell infiltrate (85). Therefore, it seems that according to the environment, CD38 can generate both immune-boosting and immune-suppressive metabolites, thereby activating or suppressing immune responses.

These at times opposing roles of CD38 in defining immune responses are in part confirmed by studies on *CD38*-deficient mice. Interestingly, when the animals are kept in clean facilities without infectious challenges, they grow and develop normally, without major defects (86). On the other hand, during infections they show impaired lymphocyte activation and homing and are ultimately more susceptible to death due to sepsis (87, 88). *CD38*-deficient animals also show reduced tumor formation, attributed to the lack CD38-mediated immunosuppression.

In the human system, CD38 is widely expressed on the surface of immune cells, particularly in conditions of cellular activation. On the cell surface, CD38 is part of the immunological synapse, forming lateral associations with critical receptors on T, B, and myeloid cells, thereby positioning itself at the center of action (48, 49). In fact, it was reported that CD38 localizes in close contact with T cell receptor (TCR), the B cell receptor (BCR), and key chemokine receptors, among other molecules (89). Perhaps the best understood function of CD38 is in the regulation of T lymphocyte functions, where the enzyme works again different ways (90–93).

First, CD38-dependent-Ca^2+^ signaling directly contributes to T cell activation, likely providing an essential second signal that drives gene expression and consequently differentiation, development, and cytotoxicity (93–95).

As a second level of T cell regulation, the NAD/CD38 axis was proposed to control T cell metabolic reprogramming needed for full T lymphocyte activation through the modulation of sirtuin activity (90, 96). Several studies are shedding light on this molecular circuit as an important metabolic checkpoint contributing to several aspects of cellular energy metabolisms, including glycolysis, oxidative phosphorylation (OXPHOS), glutaminolysis, which are strictly associated with T cell functional fate (90, 93, 97, 98). According to the models proposed, expression of CD38 on the cell surface would limit intracellular NAD levels, negatively impacting on the activities of the NAD-dependent enzymes SIRT1 and SIRT3, which are deacetylases with fundamental roles in epigenetic regulation (93). Lastly, recent data indicate that CD38 is highly expressed by specific subsets of immunosuppressive tumor infiltrating lymphocytes, including regulatory T cells and T helper 17 cells (90, 99–101). Expression of the molecule occurs often in association with exhaustion markers, such as programmed cell death protein 1 (PD-1), pointing to an active role of CD38 in modulating T cell fate toward the generation of an immune tolerant landscape in tumors, likely through the generation of ADO (90) (**Figure 3**).

What remains unclear so far is what are the factors that tip the balance in favor of Ca^2+^-active metabolites and hence immune activation or in favor of ADO and hence immunosuppression (93, 94, 102–104). Therefore, inhibition of CD38 is a valid therapeutic strategy to reestablish a functional immune surveillance (105), open the way to combination therapies with immune checkpoint inhibitors, as discussed in a separate paragraph.

## eNAD Biosynthetic-Signaling System in Regulating Immune Responses

Beside NAD-consuming, also NBEs were reported in the extracellular compartment. The best known and characterized among them is nicotidamide phosphoribosyltransferase (NAMPT), which catalyzes the conversion of Nam to NMN in the presence of phosphoribosyl pyrophosphate (PRPP) and ATP (7, 44).

The presence of NAMPT in biological fluids is now well established: however, several years were needed before realizing that a cytokine promoting B cell differentiation and originally described in mid-nineties (106), and an extracellular adipokine called visfatin were in fact the same protein as NAMPT (11, 107, 108). Of note, different cell types, including neutrophils, monocytes, macrophages, and cancer cells secrete eNAMPT in the extracellular space in response to inflammation, cellular stress, infections, and hypoxic conditions, among others. In human plasma eNAMPT normal levels are in the low nanomolar range (2-4 ng/ml), but it is over-expressed in several inflammatory and metabolic disorders, including cancer, where concentrations can increase 10-20 times (11, 108).

The second NBE dosed in biological fluids is nicotinic acid phosphoribosyltransferase (NAPRT), which controls the NAD generation pathway starting from Na. While the NAMPT pathway is probably the predominant one in most cells and tissues, considering that all NAD-consuming enzymes generate Nam, the activity of NAPRT is believed to boost NAD levels in stress conditions (44, 109–111). Information on eNAPRT is far more limited, even though concentration data indicate again a physiological level in low nanomolar range (1-2 ng/ml), raising sometimes dramatically, particularly during sepsis (112).

Whether these enzymes are active in the extracellular compartment remains uncertain, mainly because of the absence of detectable PRPP levels, an essential co-factor to produce NMN and nicotinic acid mononucleotide (NaMN). In addition, the rest of the enzymatic cascade producing NAD has never been reported in the extracellular space (109). From data present in the literature, we can exclude a direct eNAD synthesis in physiological conditions, but we cannot exclude a site-specific and transient eNAD synthesis in inflammatory conditions, due to release of intracellular molecules (ATP, PRPP) and enzymes. In favor of a compartment-specific function, the active forms of these enzymes are in a dimeric conformation, but within the extracellular compartment they should be in a monomeric, and hence inactive, form (113). Lastly, functional studies have shown that eNAMPT and eNAPRT, genetically modified to be enzymatically inactive, retain their pro-inflammatory properties (112) (**Figure 3**).

A second area of investigation concerns the mechanisms of trafficking of these enzymes from the intracellular to the extracellular space, which appear “non-classical”, as secretion is unaffected by monensin and brefeldin A, two inhibitors of the classical endoplasmatic reticulum (ER)–Golgi secretory pathway (114–117). An interesting finding indicates that NAMPT secretion could be regulated through SIRT1- and SIRT6-deacetylation, thereby linking NAD-biosynthetic and -consuming enzymes, and potentially suggesting eNAMPT secretion as regulatory mechanism to decrease its intracellular concentrations (118, 119). Recent evidence showed that eNAMPT is carried in extracellular vesicles (EVs) through systemic circulation in mice and humans. EV-contained-eNAMPT is internalized into cells, enhancing NMN and hence NAD synthesis (120). eNAMPT is actively secreted *via* exosomes also from microglia during neuroinflammation due to ischemic injury (121). These findings support the possibility of metabolic exchange between tumor/inflammatory and immune cells and vice versa within the site of inflammation or the tumor microenvironment (TME), as previously described for other cytokines and metabolic molecules (122, 123).

The conclusion from these data is that outside of cells it is unlikely that NAMPT and NAPRT function as NAD-producing enzymes, raising the alternative possibility that they possess different functions. In fact, eNAMPT can directly bind Toll-like receptor 4 (TLR4) (112, 124) and C–C chemokine receptor type 5 (CCR5) (125), which might explain how the protein is involved in the activation of an inflammatory signature. The binding with TLR4 was demonstrated in different cellular models, leading to activation of specific intracellular signaling pathways (e.g., STAT3, NF-κB, Akt, P38) within minutes, and activation of inflammasome in few hours (112, 124).

Less recently, it was reported that eNAMPT can selectively inhibit infection of monocytes by human immunodeficiency virus (HIV) and this activity was linked to a direct interaction with CCR5, shown using surface plasmon resonance (SPR) (126). More recently, Torretta et al. suggested that eNAMPT acts as a natural antagonist of CCR5 in cancer cells (125). Within the cancer microenvironment, eNAMPT seems to contribute to shape an immunotolerant environment, mostly acting on the myeloid component. We described a role for eNAMPT in the differentiation of circulating monocytes from chronic lymphocytic leukemia (CLL) patients toward tumor-supporting M2 macrophages (127). Recently, it was demonstrated that iNAMPT acts also on myeloid-derived suppressor cells (MDSCs) *via* a SIRT1/hypoxia-inducible factor (HIF)-1α axis, promoting their mobilization (128). The activation of these circuits creates an immunosuppressive and tumor-promoting microenvironment (**Figure 3**).

Much less is known on eNAPRT, even though from early information it seems to possess properties similar to NAMPT when in extracellular fluids. Managò et al. demonstrated that eNAPRT binds TLR4 on macrophages triggering NF-kB activation and pro-inflammatory cytokines secretion (112). Moreover, eNAPRT shares with eNAMPT the activation of a transcriptional program, maybe mediated by the induction of macrophage colony-stimulating factor (M-CFS), to force monocyte differentiation into macrophages. In turn, macrophages are a source of eNAMPT and eNAPRT *in vivo* (112). Even if several issues remain to be investigated, a functional role of these enzymes in primary innate immunity responses is clearly emerging, opening the way to target these enzymes to modulate inflammation.

## Is the NADome a Therapeutic Target?

Alterations in the NADome have been described in several human diseases, including inflammatory conditions (gastric and intestinal inflammatory diseases, graft-versus-host disease, sepsis and multiple organ failure, allergies particularly in the lungs, atherosclerosis, age-associated insulin resistance, neuroinflammation/degeneration), autoimmune diseases (multiple sclerosis, psoriasis, systemic lupus erithematosous), cardiovascular diseases and cancer (7, 55, 129).

**Figure 4:**
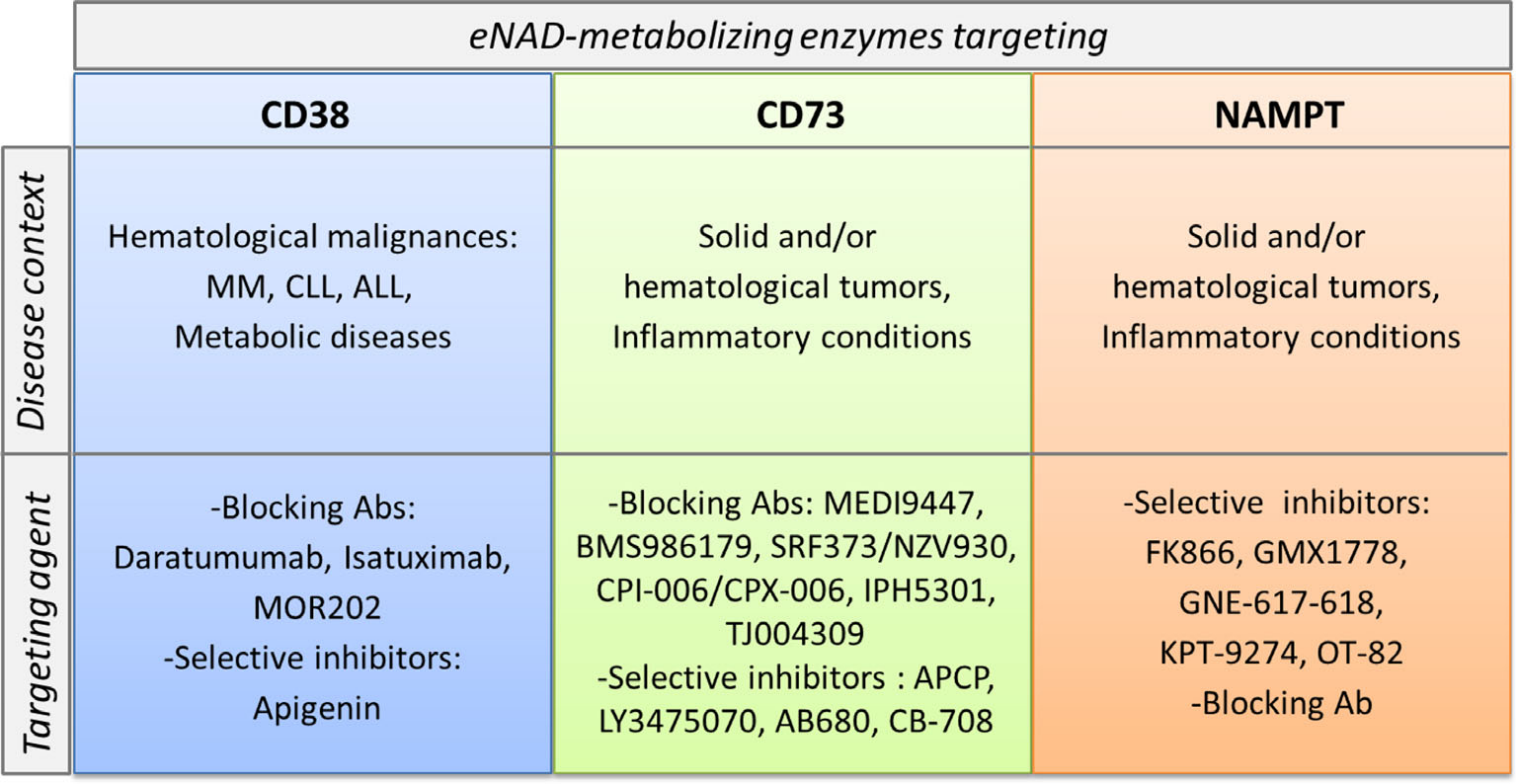
CD38, CD73 and NAMPT as markers and therapeutic targets in pathological conditions. CD38, CD73 and NAMPT expression levels increase in several pathological conditions. These molecules become markers of aggressive disease. In the lower part of the cartoon specific pharmacological inhibitors and/or blocking antibodies, currently in preclinical or clinical trials for each target are listed.

In addition to their role in shaping the immune system and in creating immunosuppressive conditions, in some instances NAD-metabolizing enzymes are considered biological prognostic markers and therapeutic targets. Among them, the most promising are CD38, CD73 and NAMPT and the disease setting is cancer (**Figure 4**).

CD38 is expressed in hematological malignancies, including acute B lymphoblastic leukemia (B-ALL), acute myeloid leukemia (AML), mantle cell lymphoma (MCL), CLL, multiple myeloma (MM) and NK/T cell leukemia (T-ALL) (55, 94, 102, 105, 130–132). The role of CD38 has been widely explored and defined in CLL and in MM. On CLL B lymphocytes, CD38 associates with the BCR complex [BCR/CD81/CD19/CD21] and cooperates to amplify the signal transduction driving cell proliferation (55, 133, 134).

Patients with CLL with a higher proportion of leukemic cells expressing CD38 ≥30% experience a shorter time to first treatment and a more aggressive clinical course with inferior overall survival compared to patients who have <30% of CD38^+^ CLL cells, thus establishing surface CD38 as a marker of poor prognosis (135–137).

MM is a plasma cell neoplastic aggressive disease with a median overall survival of 4.4–7.1 years (138). CD38 is highly and ubiquitously expressed on MM cells and at low levels on normal lymphoid and myeloid cells (49, 139). Daratumumab is a first-in-class anti-CD38 therapeutic monoclonal antibody (mAb) approved in 2015 for the treatment of relapsed/refractory MM (140). The documented mechanisms of action include antibody-dependent cell cytotoxicity (ADCC), complement dependent cytotoxicity (CDC), antibody-dependent cellular phagocytosis (ADCP), and inhibition of CD38 enzymatic activities and induction of apoptosis in a caspase-dependent manner (132, 141, 142). This Ab is now used in combination with other drugs; however, the density of CD38 molecules on MM cells is a predictive factor to the efficacy and durability of daratumumab treatment (143). In CLL, CD38 engagement by daratumumab modulates BCR signaling and enhances the anti-CLL activity of ibrutinib, an inhibitor of BCR signaling (144). In addition, CD38 is highly expressed in different solid tumors (i.e., gliomas, pancreatic cancer, non-small cell lung cancer, melanoma, hepatocellular carcinoma), generally associated to increased aggressiveness and creating a tumor-supporting microenvironment (145), providing a rationale for the expansion of daratumumab’s field of action.

Targeting CD73 to interfere with the degradation of AMP into ADO, reducing the generation of an immunosuppressed and pro-angiogenic niche that promotes the onset and progression of cancer, is an attractive therapeutic option (146). CD73 expression is higher in the majority of human solid tumors. Its expression and activity are closely associated with tumor invasiveness and metastasis (147, 148).

Inhibition of CD73 using either mAb or small molecule inhibitors such as a,b-methylene-ADP (APCP) have demonstrated antitumor activities in preclinical tumor mouse models (148, 149). Furthermore, a number of anti-CD73 mAbs (MEDI9447, BMS986179, SRF373/NZV930, CPI-006/CPX-006, IPH5301, TJ004309) and selective inhibitors (LY3475070, AB680, CB-708) are being tested in early phase clinical trials, as recently reviewed in (147, 150).

Therefore, combination therapies with CD73 blocking Abs or small molecule inhibitors and other therapeutic strategies including immune checkpoint blockade, adoptive T cell therapy, agonistic immunotherapy, chemotherapy, and radiation therapy, could have synergic effects in various cancers boosting immune response to keep the tumor cells in control, as emerged by recent studies (148, 151).

The first NAMPT inhibitor FK866 (also known as APO866) was described in 2003 by Hasmann et al. (152) Since that, several specific NAMPT inhibitors were developed as recently reviewed in in (7, 153, 154). The rationale was mainly supported by the over-expression of NAMPT in cancer cells, as extensively described by us and by several research groups (11, 108, 117, 127, 155–158).

This led to a first wave of molecules that entered clinical trials for cancer therapy; however, no molecules reported to have progressed to later stages [www.clinicaltrials.gov (7, 153)].

Toxicity of old inhibitors and rescue mechanisms by the activation of other NBEs following NAMPT block, have limited the use of NAMPT inhibitors as single agents. However, increasing evidence suggests that a better selection of tumor subtype rely exclusively on NAMPT activity to generate NAD, as well as novel drugs less toxic, could open a second life for NAMPT inhibition strategy. Moreover, a combination between NAMPT inhibitors and selective inhibitors of oncogenic signaling driving cancer progression could be therapeutically exploited as suggested (11, 117, 159).

An unknown notion is whether these inhibitors could also affect eNAMPT activity, even if, as mentioned before, the enzymatic activity of eNAMPT is controversial. Travelli et al. developed novel inhibitors that can’t cross the plasma membrane and have more activity to block eNAMPT form, demonstrating reduced growth of triple negative mammary carcinoma in mice (160). On the other hand, there is also intense research to develop a blocking antibody to neutralize eNAMPT and reduce its “cytokine-like activity” within the TME. The group of Garcia firstly has devised a polyclonal eNAMPT neutralizing antibody (pAb) (161). They used this Ab in different models of inflammation and cancer, including lung injury and prostate cancer. Recently, in acute respiratory distress syndrome (ARDS) they demonstrated the highly significant contribution of endothelial cell (EC)-derived NAMPT to the severity of inflammatory lung injury in preclinical ARDS models. Intravenous delivery of either eNAMPT-neutralizing pAb/mAb significantly attenuated inflammatory lung injury in mouse model. *In vitro* studies on EC demonstrated that eNAMPT-neutralizing antibodies strongly abrogate eNAMPT-induced TLR4 pathway activation (162). In invasive prostate cancer (PCa) Sun et al. proved the activity of eNAMPT in supporting the invasive features of PCa, and the tumor blocking activity of the anti-eNAMPT neutralizing antibody in a pre-clinical *in vivo* model of PCa invasion (163). In parallel, the group of Prof. Genazzani in Italy is developing a novel monoclonal antibody (C269) that neutralizes *in vitro* the cytokine-like action of eNAMPT and that reduces its serum levels in rodents. This Ab is able to significantly reduce acute and chronic colitis in two models of induced-colitis (164), suggesting a role of eNAMPT in the pathogenesis of inflammatory bowel disease (IBD) and the therapeutic potential of its neutralization in this pathology. The general idea of targeting eNAMPT in tumors and in inflammatory diseases is increasing to counteract the extracellular functions of this protein, mainly linked to the activation of TLR4 and modulation of immune responses. The best option could be to combine i/eNAMPT targeting with immunomodulatory agents to obtain a tumor growth regression and a concomitant reversion of immunosuppressive conditions, acting on the immune system. In support of this, two papers demonstrated that NAMPT inhibitors enhance the anti-tumor efficacy of immune checkpoint inhibitors, i.e. antibody against PD-1 (128, 165).

## Conclusions and Future Perspectives

Since the discovery of the presence of extracellular nucleotides such as ATP and NAD released from intracellular stores in conditions of cell stress or inflammation, they are considered “danger signals” to alert the immune system, participating in the recruitment, activation, and differentiation of immune cells, and promoting the production and release of pro-inflammatory cytokines. Within the TME, extracellular nucleotides create pro-tumor conditions acting directly on tumor aggressive features but also on immune cells promoting a general immunosuppression.

The extracellular machinery that regulates eNAD functions is complex, as we summarized in this review several eNAD-metabolizing enzymes rapidly degrade it into metabolites that in turn can function as signaling messengers or can be internalized and used to reconstitute the intracellular NAD pool. Directly, eNAD can bind purinergic receptors and activate signaling. The effects of eNAD are therefore dependent on the presence of receptors, metabolizing enzymes and cellular stress conditions within the microenvironment. Understanding this intricate machinery remains the most important challenge to develop therapeutic strategies to modulate expression of these extracellular nucleotides, relative enzymes, and receptors to re-educate the immune system in different diseases, including cancer.

## Author Contributions

SD designed and reviewed the work, which was assembled by VA, with contribution of VM and LB. All authors contributed to the article and approved the submitted version.

## Funding

This work was supported by the Ministry of Education University and Research-MIUR, PRIN Project 2017CBNCYT and Progetto strategico di Eccellenza Dipartimentale #D15D18000410001 (the latter awarded to the Dept. of Medical Sciences, University of Turin) and ITN INTEGRATA program (grant agreement 813284), and by Associazione Italiana Ricerca sul Cancro (AIRC), Investigator Grant –IG 2019 #23095.

## Conflict of Interest

The authors declare that the research was conducted in the absence of any commercial or financial relationships that could be construed as a potential conflict of interest.

## Publisher’s Note

All claims expressed in this article are solely those of the authors and do not necessarily represent those of their affiliated organizations, or those of the publisher, the editors and the reviewers. Any product that may be evaluated in this article, or claim that may be made by its manufacturer, is not guaranteed or endorsed by the publisher.

## References

[1] Vander Heiden MG Cantley LC Thompson CB. Understanding the Warburg Effect: The Metabolic Requirements of Cell Proliferation. Science (2009) 324(5930):1029–33. 10.1126/science.1160809 PMC2849637 19460998

[2] Bonora M Patergnani S Rimessi A De Marchi E Suski JM Bononi A. ATP Synthesis and Storage. Purinergic Signal (2012) 8(3):343–57. 10.1007/s11302-012-9305-8 PMC3360099 22528680

[3] Xiao W Wang RS Handy DE Loscalzo J. NAD(H) and NADP(H) Redox Couples and Cellular Energy Metabolism. Antioxid Redox Signal (2018) 28(3):251–72. 10.1089/ars.2017.7216 PMC5737637 28648096

[4] Yaku K Okabe K Nakagawa T. NAD Metabolism: Implications in Aging and Longevity. Ageing Res Rev (2018) 47:1–17. 10.1016/j.arr.2018.05.006 29883761

[5] Linden J Koch-Nolte F Dahl G. Purine Release, Metabolism, and Signaling in the Inflammatory Response. Annu Rev Immunol (2019) 37:325–47. 10.1146/annurev-immunol-051116-052406 30676821

[6] Amjad S Nisar S Bhat AA Shah AR Frenneaux MP Fakhro K. Role of NAD(+) in Regulating Cellular and Metabolic Signaling Pathways. Mol Metab (2021) 49:101195. 10.1016/j.molmet.2021.101195 33609766 PMC7973386

[7] Xie N Zhang L Gao W Huang C Huber PE Zhou X. NAD(+) Metabolism: Pathophysiologic Mechanisms and Therapeutic Potential. Signal Transduct Target Ther (2020) 5(1):227. 10.1038/s41392-020-00311-7 33028824 PMC7539288

[8] Di Virgilio F Sarti AC Falzoni S De Marchi E Adinolfi E. Extracellular ATP and P2 Purinergic Signalling in the Tumour Microenvironment. Nat Rev Cancer (2018) 18(10):601–18. 10.1038/s41568-018-0037-0 30006588

[9] Haag F Adriouch S Brass A Jung C Moller S Scheuplein F. Extracellular NAD and ATP: Partners in Immune Cell Modulation. Purinergic Signal (2007) 3(1-2):71–81. 10.1007/s11302-006-9038-7 18404420 PMC2096762

[10] Scheuplein F Schwarz N Adriouch S Krebs C Bannas P Rissiek B. NAD+ and ATP Released From Injured Cells Induce P2X7-Dependent Shedding of CD62L and Externalization of Phosphatidylserine by Murine T Cells. J Immunol (2009) 182(5):2898–908. 10.4049/jimmunol.0801711 19234185

[11] Audrito V Messana VG Deaglio S. NAMPT and NAPRT: Two Metabolic Enzymes With Key Roles in Inflammation. Front Oncol (2020) 10:358. 10.3389/fonc.2020.00358 32266141 PMC7096376

[12] Pellegatti P Raffaghello L Bianchi G Piccardi F Pistoia V Di Virgilio F. Increased Level of Extracellular ATP at Tumor Sites: *In Vivo* Imaging With Plasma Membrane Luciferase. PloS One (2008) 3(7):e2599. 10.1371/journal.pone.0002599 18612415 PMC2440522

[13] Wilhelm K Ganesan J Muller T Durr C Grimm M Beilhack A. Graft-Versus-Host Disease Is Enhanced by Extracellular ATP Activating P2X7R. Nat Med (2010) 16(12):1434–8. 10.1038/nm.2242 21102458

[14] O’Reilly T Niven DF. Levels of Nicotinamide Adenine Dinucleotide in Extracellular Body Fluids of Pigs may be Growth-Limiting for Actinobacillus Pleuropneumoniae and Haemophilus Parasuis. Can J Vet Res (2003) 67(3):229–31. PMC227058 12889731

[15] Billington RA Bruzzone S De Flora A Genazzani AA Koch-Nolte F Ziegler M. Emerging Functions of Extracellular Pyridine Nucleotides. Mol Med (2006) 12(11-12):324–7. 10.2119/2006-00075.Billington PMC1829198 17380199

[16] Di Stefano M Conforti L. Diversification of NAD Biological Role: The Importance of Location. FEBS J (2013) 280(19):4711–28. 10.1111/febs.12433 23848828

[17] Trautmann A. Extracellular ATP in the Immune System: More Than Just a “Danger Signal”. Sci Signal (2009) 2(56):pe6. 10.1126/scisignal.256pe6 19193605

[18] Corriden R Insel PA. Basal Release of ATP: An Autocrine-Paracrine Mechanism for Cell Regulation. Sci Signal (2010) 3(104):re1. 10.1126/scisignal.3104re1 20068232 PMC3085344

[19] Schilling E Hauschildt S. Extracellular ATP Induces P2X7-Dependent Nicotinamide Phosphoribosyltransferase Release in LPS-Activated Human Monocytes. Innate Immun (2012) 18(5):738–44. 10.1177/1753425912439614 22377803

[20] Adriouch S Haag F Boyer O Seman M Koch-Nolte F. Extracellular NAD(+): A Danger Signal Hindering Regulatory T Cells. Microbes Infect (2012) 14(14):1284–92. 10.1016/j.micinf.2012.05.011 22634347

[21] Bianchi ME. DAMPs, PAMPs and Alarmins: All We Need to Know About Danger. J Leukoc Biol (2007) 81(1):1–5. 10.1189/jlb.0306164 17032697

[22] Venereau E Ceriotti C Bianchi ME. DAMPs From Cell Death to New Life. Front Immunol (2015) 6:422. 10.3389/fimmu.2015.00422 26347745 PMC4539554

[23] Tanaka K Choi J Cao Y Stacey G. Extracellular ATP Acts as a Damage-Associated Molecular Pattern (DAMP) Signal in Plants. Front Plant Sci (2014) 5:446. 10.3389/fpls.2014.00446 25232361 PMC4153020

[24] Di Virgilio F Dal Ben D Sarti AC Giuliani AL Falzoni S. The P2X7 Receptor in Infection and Inflammation. Immunity (2017) 47(1):15–31. 10.1016/j.immuni.2017.06.020 28723547

[25] Smyth LM Bobalova J Mendoza MG Lew C Mutafova-Yambolieva VN. Release of Beta-Nicotinamide Adenine Dinucleotide Upon Stimulation of Postganglionic Nerve Terminals in Blood Vessels and Urinary Bladder. J Biol Chem (2004) 279(47):48893–903. 10.1074/jbc.M407266200M407266200[pii 15364945

[26] Breen LT Smyth LM Yamboliev IA Mutafova-Yambolieva VN. Beta-NAD Is a Novel Nucleotide Released on Stimulation of Nerve Terminals in Human Urinary Bladder Detrusor Muscle. Am J Physiol Renal Physiol (2006) 290(2):F486–95. 10.1152/ajprenal.00314.2005 16189287

[27] Mutafova-Yambolieva VN Hwang SJ Hao X Chen H Zhu MX Wood JD. Beta-Nicotinamide Adenine Dinucleotide Is an Inhibitory Neurotransmitter in Visceral Smooth Muscle. Proc Natl Acad Sci USA (2007) 104(41):16359–64. 10.1073/pnas.0705510104 PMC2042211 17913880

[28] Adriouch S Hubert S Pechberty S Koch-Nolte F Haag F Seman M. NAD(+) Released During Inflammation Participates in T Cell Homeostasis by Inducing ART2-Mediated Death of Naive T Cells *In Vivo* . J Immunol (2007) 179(1):186–94. 10.4049/jimmunol.179.1.186 17579037

[29] Cekic C Linden J. Purinergic Regulation of the Immune System. Nat Rev Immunol (2016) 16(3):177–92. 10.1038/nri.2016.4 26922909

[30] Bruzzone S Guida L Zocchi E Franco L De Flora A. Connexin 43 Hemi Channels Mediate Ca2+-Regulated Transmembrane NAD+ Fluxes in Intact Cells. FASEB J (2001) 15(1):10–2. 10.1096/fj.00-0566fje 11099492

[31] Hwang SJ Durnin L Dwyer L Rhee PL Ward SM Koh SD. Beta-Nicotinamide Adenine Dinucleotide Is an Enteric Inhibitory Neurotransmitter in Human and Nonhuman Primate Colons. Gastroenterology (2011) 140(2):608–17 e6. 10.1053/j.gastro.2010.09.039 20875415 PMC3031738

[32] Mottahedeh J Haffner MC Grogan TR Hashimoto T Crowell PD Beltran H. CD38 is Methylated in Prostate Cancer and Regulates Extracellular NAD(). Cancer Metab (2018) 6:13. 10.1186/s40170-018-0186-3 30258629 PMC6150989

[33] Sitkovsky MV Ohta A. The ‘Danger’ Sensors That STOP the Immune Response: The A2 Adenosine Receptors? Trends Immunol (2005) 26(6):299–304. 10.1016/j.it.2005.04.004 15922945

[34] Deaglio S Robson SC. Ectonucleotidases as Regulators of Purinergic Signaling in Thrombosis, Inflammation, and Immunity. Adv Pharmacol (2011) 61:301–32. 10.1016/B978-0-12-385526-8.00010-2 PMC5879773 21586363

[35] Vaisitti T Audrito V Serra S Bologna C Brusa D Malavasi F. NAD+-Metabolizing Ecto-Enzymes Shape Tumor-Host Interactions: The Chronic Lymphocytic Leukemia Model. FEBS Lett (2011) 585(11):1514–20. 10.1016/j.febslet.2011.04.036 21514298

[36] Kazemi MH Raoofi Mohseni S Hojjat-Farsangi M Anvari E Ghalamfarsa G Mohammadi H. Adenosine and Adenosine Receptors in the Immunopathogenesis and Treatment of Cancer. J Cell Physiol (2018) 233(3):2032–57. 10.1002/jcp.25873 28233320

[37] Di Virgilio F. Purines, Purinergic Receptors, and Cancer. Cancer Res (2012) 72(21):5441–7. 10.1158/0008-5472.CAN-12-16000008-5472.CAN-12-1600[pii 23090120

[38] Di Virgilio F Sarti AC Coutinho-Silva R. Purinergic Signaling, DAMPs, and Inflammation. Am J Physiol Cell Physiol (2020) 318(5):C832–C5. 10.1152/ajpcell.00053.2020 32159362

[39] Gasparrini M Sorci L Raffaelli N. Enzymology of Extracellular NAD Metabolism. Cell Mol Life Sci (2021) 78(7):3317–31. 10.1007/s00018-020-03742-1 PMC8038981 33755743

[40] Audrito V Messana VG Moiso E Vitale N Arruga F Brandimarte L. NAMPT Over-Expression Recapitulates the BRAF Inhibitor Resistant Phenotype Plasticity in Melanoma. Cancers (Basel) (2020) 12(12):3855–77. 10.3390/cancers12123855 PMC7766175 33419372

[41] Houtkooper RH Canto C Wanders RJ Auwerx J. The Secret Life of NAD+: An Old Metabolite Controlling New Metabolic Signaling Pathways. Endocr Rev (2010) 31(2):194–223. 10.1210/er.2009-0026 20007326 PMC2852209

[42] Hassinen IE. Signaling and Regulation Through the NAD(+) and NADP(+) Networks. Antioxid Redox Signal (2019) 30(6):857–74. 10.1089/ars.2017.7479 29284289

[43] Nikiforov A Dolle C Niere M Ziegler M. Pathways and Subcellular Compartmentation of NAD Biosynthesis in Human Cells: From Entry of Extracellular Precursors to Mitochondrial NAD Generation. J Biol Chem (2011) 286(24):21767–78. 10.1074/jbc.M110.213298M110.213298[pii PMC3122232 21504897

[44] Ruggieri S Orsomando G Sorci L Raffaelli N. Regulation of NAD Biosynthetic Enzymes Modulates NAD-Sensing Processes to Shape Mammalian Cell Physiology Under Varying Biological Cues. Biochim Biophys Acta (2015) 1854(9):1138–49. 10.1016/j.bbapap.2015.02.021 25770681

[45] Canto C Menzies KJ Auwerx J. NAD(+) Metabolism and the Control of Energy Homeostasis: A Balancing Act Between Mitochondria and the Nucleus. Cell Metab (2015) 22(1):31–53. 10.1016/j.cmet.2015.05.023 26118927 PMC4487780

[46] Grolla AA Miggiano R Di Marino D Bianchi M Gori A Orsomando G. A Nicotinamide Phosphoribosyltransferase-GAPDH Interaction Sustains the Stress-Induced NMN/NAD(+) Salvage Pathway in the Nucleus. J Biol Chem (2020) 295(11):3635–51. 10.1074/jbc.RA119.010571 PMC7076215 31988240

[47] Nikiforov A Kulikova V Ziegler M. The Human NAD Metabolome: Functions, Metabolism and Compartmentalization. Crit Rev Biochem Mol Biol (2015) 50(4):284–97. 10.3109/10409238.2015.1028612 PMC4673589 25837229

[48] Chini EN. CD38 as a Regulator of Cellular NAD: A Novel Potential Pharmacological Target for Metabolic Conditions. Curr Pharm Des (2009) 15(1):57–63. 10.2174/138161209787185788 19149603 PMC2883294

[49] Malavasi F Deaglio S Funaro A Ferrero E Horenstein AL Ortolan E. Evolution and Function of the ADP Ribosyl Cyclase/CD38 Gene Family in Physiology and Pathology. Physiol Rev (2008) 88(3):841–86. 10.1152/physrev.00035.2007 18626062

[50] Deaglio S Malavasi F. The CD38/CD157 Mammalian Gene Family: An Evolutionary Paradigm for Other Leukocyte Surface Enzymes. Purinergic Signaling (2006) 2:431–41. 10.1007/s11302-006-9002-6 PMC2096639 18404481

[51] Seman M Adriouch S Haag F Koch-Nolte F. Ecto-ADP-Ribosyltransferases (ARTs): Emerging Actors in Cell Communication and Signaling. Curr Med Chem (2004) 11(7):857–72. 10.2174/0929867043455611 15078170

[52] Katada T Kontani K Wada T Hosoda N Hoshino S Nishina H. Enzymic and Signal Transduction Properties of CD38/NADase and PC-1/Phosphodiesterase. Chem Immunol (2000) 75:60–78. 10.1159/000058762 10851779

[53] Garavaglia S Bruzzone S Cassani C Canella L Allegrone G Sturla L. The High-Resolution Crystal Structure of Periplasmic Haemophilus Influenzae NAD Nucleotidase Reveals a Novel Enzymatic Function of Human CD73 Related to NAD Metabolism. Biochem J (2012) 441(1):131–41. 10.1042/BJ20111263 21933152

[54] Horenstein AL Chillemi A Zaccarello G Bruzzone S Quarona V Zito A. A CD38/CD203a/CD73 Ectoenzymatic Pathway Independent of CD39 Drives a Novel Adenosinergic Loop in Human T Lymphocytes. Oncoimmunology (2013) 2(9):e26246. 10.4161/onci.26246 24319640 PMC3850273

[55] Vaisitti T Arruga F Guerra G Deaglio S. Ectonucleotidases in Blood Malignancies: A Tale of Surface Markers and Therapeutic Targets. Front Immunol (2019) 10:2301. 10.3389/fimmu.2019.02301 31636635 PMC6788384

[56] Kemmer G Reilly TJ Schmidt-Brauns J Zlotnik GW Green BA Fiske MJ. NadN and E (P4) are Essential for Utilization of NAD and Nicotinamide Mononucleotide But Not Nicotinamide Riboside in Haemophilus Influenzae. J Bacteriol (2001) 183(13):3974–81. 10.1128/JB.183.13.3974-3981.2001 PMC95280 11395461

[57] Grozio A Sociali G Sturla L Caffa I Soncini D Salis A. CD73 Protein as a Source of Extracellular Precursors for Sustained NAD+ Biosynthesis in FK866-Treated Tumor Cells. J Biol Chem (2013) 288(36):25938–49. 10.1074/jbc.M113.470435M113.470435 PMC3764798 23880765

[58] Sociali G Raffaghello L Magnone M Zamporlini F Emionite L Sturla L. Antitumor Effect of Combined NAMPT and CD73 Inhibition in an Ovarian Cancer Model. Oncotarget (2015) 7(3):2968–84. 10.18632/oncotarget.65026502 PMC4823084 26658104

[59] Burnstock G. Purinergic Nerves. Pharmacol Rev (1972) 24(3):509–81. 4404211

[60] Burnstock G Verkhratsky A. Evolutionary Origins of the Purinergic Signalling System. Acta Physiol (Oxf) (2009) 195(4):415–47. 10.1111/j.1748-1716.2009.01957.x 19222398

[61] Burnstock G. Purine and Purinergic Receptors. Brain Neurosci Adv (2018) 2:2398212818817494. 10.1177/2398212818817494 32166165 PMC7058212

[62] Moreschi I Bruzzone S Nicholas RA Fruscione F Sturla L Benvenuto F. Extracellular NAD+ Is an Agonist of the Human P2Y11 Purinergic Receptor in Human Granulocytes. J Biol Chem (2006) 281(42):31419–29. 10.1074/jbc.M606625200 16926152

[63] Klein C Grahnert A Abdelrahman A Muller CE Hauschildt S. Extracellular NAD(+) Induces a Rise in [Ca(2+)](i) in Activated Human Monocytes *via* Engagement of P2Y(1) and P2Y(11) Receptors. Cell Calcium (2009) 46(4):263–72. 10.1016/j.ceca.2009.08.004 19748117

[64] Alefishat E Alexander SP Ralevic V. Effects of NAD at Purine Receptors in Isolated Blood Vessels. Purinergic Signal (2015) 11(1):47–57. 10.1007/s11302-014-9428-1 25315718 PMC4336311

[65] Durnin L Hwang SJ Ward SM Sanders KM Mutafova-Yambolieva VN. Adenosine 5-Diphosphate-Ribose Is a Neural Regulator in Primate and Murine Large Intestine Along With Beta-NAD(+). J Physiol (2012) 590(Pt 8):1921–41. 10.1113/jphysiol.2011.222414 PMC3573313 22351627

[66] Grahnert A Klein C Hauschildt S. Involvement of P2X Receptors in the NAD(+)-Induced Rise in [Ca (2+)] (I) in Human Monocytes. Purinergic Signal (2009) 5(3):309–19. 10.1007/s11302-009-9144-4 PMC2717312 19221895

[67] Khakh BS North RA. P2X Receptors as Cell-Surface ATP Sensors in Health and Disease. Nature (2006) 442(7102):527–32. 10.1038/nature04886 16885977

[68] Dubyak GR. P2X7 Receptor Regulation of Non-Classical Secretion From Immune Effector Cells. Cell Microbiol (2012) 14(11):1697–706. 10.1111/cmi.12001 PMC3473166 22882764

[69] Adinolfi E Giuliani AL De Marchi E Pegoraro A Orioli E Di Virgilio F. The P2X7 Receptor: A Main Player in Inflammation. Biochem Pharmacol (2018) 151:234–44. 10.1016/j.bcp.2017.12.021 29288626

[70] Giuliani AL Sarti AC Falzoni S Di Virgilio F. The P2X7 Receptor-Interleukin-1 Liaison. Front Pharmacol (2017) 8:123. 10.3389/fphar.2017.00123 28360855 PMC5353276

[71] Seman M Adriouch S Scheuplein F Krebs C Freese D Glowacki G. NAD-Induced T Cell Death: ADP-Ribosylation of Cell Surface Proteins by ART2 Activates the Cytolytic P2X7 Purinoceptor. Immunity (2003) 19(4):571–82. 10.1016/S1074-7613(03)00266-8 14563321

[72] Hubert S Rissiek B Klages K Huehn J Sparwasser T Haag F. Extracellular NAD+ Shapes the Foxp3+ Regulatory T Cell Compartment Through the ART2-P2X7 Pathway. J Exp Med (2010) 207(12):2561–8. 10.1084/jem.20091154 PMC2989765 20975043

[73] Alessio M Roggero S Funaro A De Monte LB Peruzzi L Geuna M. CD38 Molecule: Structural and Biochemical Analysis on Human T Lymphocytes, Thymocytes, and Plasma Cells. J Immunol (1990) 145(3):878–84. 1695648

[74] Malavasi F Deaglio S Ferrero E Funaro A Sancho J Ausiello CM. CD38 and CD157 as Receptors of the Immune System: A Bridge Between Innate and Adaptive Immunity. Mol Med (2006) 12(11-12):334–41. 10.2119/2006-00094.Malavasi PMC1829205 17380201

[75] De Flora A Guida L Franco L Zocchi E. The CD38/cyclic ADP-Ribose System: A Topological Paradox. Int J Biochem Cell Biol (1997) 29(10):1149–66. 10.1016/s1357-2725(97)00062-9 9438379

[76] Zhao YJ Lam CM Lee HC. The Membrane-Bound Enzyme CD38 Exists in Two Opposing Orientations. Sci Signal (2012) 5(241):ra67. 10.1126/scisignal.2002700 22969159

[77] Shrimp JH Hu J Dong M Wang BS MacDonald R Jiang H. Revealing CD38 Cellular Localization Using a Cell Permeable, Mechanism-Based Fluorescent Small-Molecule Probe. J Am Chem Soc (2014) 136(15):5656–63. 10.1021/ja411046j PMC4004212 24660829

[78] Liu J Zhao YJ Li WH Hou YN Li T Zhao ZY. Cytosolic Interaction of Type III Human CD38 With CIB1 Modulates Cellular Cyclic ADP-Ribose Levels. Proc Natl Acad Sci U.S.A. (2017) 114(31):8283–8. 10.1073/pnas.1703718114 PMC5547619 28720704

[79] Sumoza-Toledo A Penner R. TRPM2: A Multifunctional Ion Channel for Calcium Signalling. J Physiol (2011) 589(Pt 7):1515–25. 10.1113/jphysiol.2010.201855 PMC3099011 21135052

[80] Lee HC. Structure and Enzymatic Functions of Human CD38. Mol Med (2006) 12(11-12):317–23. 10.2119/2006-00086.Lee PMC1829193 17380198

[81] Adebanjo OA Anandatheerthavarada HK Koval AP Moonga BS Biswas G Sun L. A New Function for CD38/ADP-Ribosyl Cyclase in Nuclear Ca2+ Homeostasis. Nat Cell Biol (1999) 1(7):409–14. 10.1038/15640 10559984

[82] Guse AH. Cyclic ADP-Ribose: A Novel Ca2+-Mobilising Second Messenger. Cell Signal (1999) 11(5):309–16. 10.1016/S0898-6568(99)00004-2 10376802

[83] Colgan SP Eltzschig HK Eckle T Thompson LF. Physiological Roles for Ecto-5’-Nucleotidase (CD73). Purinergic Signal (2006) 2(2):351–60. 10.1007/s11302-005-5302-5 PMC2254482 18404475

[84] Allard D Allard B Gaudreau PO Chrobak P Stagg J. CD73-Adenosine: A Next-Generation Target in Immuno-Oncology. Immunotherapy (2016) 8(2):145–63. 10.2217/imt.15.106 26808918

[85] Chen Z Han ZC. STAT3: A Critical Transcription Activator in Angiogenesis. Med Res Rev (2008) 28(2):185–200. 10.1002/med.20101 17457812

[86] Fukushi Y Kato I Takasawa S Sasaki T Ong BH Sato M. Identification of Cyclic ADP-Ribose-Dependent Mechanisms in Pancreatic Muscarinic Ca2+ Signaling Using CD38 Knockout Mice. J Biol Chem (2001) 276(1):649–55. 10.1074/jbc.M004469200 11001947

[87] Partida-Sanchez S Cockayne DA Monard S Jacobson EL Oppenheimer N Garvy B. Cyclic ADP-Ribose Production by CD38 Regulates Intracellular Calcium Release, Extracellular Calcium Influx and Chemotaxis in Neutrophils and Is Required for Bacterial Clearance *In Vivo* . Nat Med (2001) 7(11):1209–16. 10.1038/nm1101-1209 11689885

[88] Partida-Sanchez S Goodrich S Kusser K Oppenheimer N Randall TD Lund FE. Regulation of Dendritic Cell Trafficking by the ADP-Ribosyl Cyclase CD38: Impact on the Development of Humoral Immunity. Immunity (2004) 20(3):279–91. 10.1016/S1074-7613(04)00048-2 15030772

[89] Munoz P Mittelbrunn M de la Fuente H Perez-Martinez M Garcia-Perez A Ariza-Veguillas A. Antigen-Induced Clustering of Surface CD38 and Recruitment of Intracellular CD38 to the Immunologic Synapse. Blood (2008) 111(7):3653–64. 10.1182/blood-2007-07-101600 18212246

[90] Chatterjee S Daenthanasanmak A Chakraborty P Wyatt MW Dhar P Selvam SP. CD38-NAD(+)Axis Regulates Immunotherapeutic Anti-Tumor T Cell Response. Cell Metab (2018) 27(1):85–100.e8. 10.1016/j.cmet.2017.10.006 29129787 PMC5837048

[91] Krejcik J Casneuf T Nijhof IS Verbist B Bald J Plesner T. Daratumumab Depletes CD38+ Immune Regulatory Cells, Promotes T-Cell Expansion, and Skews T-Cell Repertoire in Multiple Myeloma. Blood (2016) 128(3):384–94. 10.1182/blood-2015-12-687749 PMC4957162 27222480

[92] Sharif T Martell E Dai C Ghassemi-Rad MS Kennedy BE Lee PWK. Regulation of Cancer and Cancer-Related Genes *via* NAD. Antioxid Redox Signal (2018) 30(6):906–23. 10.1089/ars.2017.7478 29334761

[93] Kar A Mehrotra S Chatterjee S. CD38: T Cell Immuno-Metabolic Modulator. Cells (2020) 9(7):1716–36. 10.3390/cells9071716 PMC7408359 32709019

[94] Hogan KA Chini CCS Chini EN. The Multi-Faceted Ecto-Enzyme CD38: Roles in Immunomodulation, Cancer, Aging, and Metabolic Diseases. Front Immunol (2019) 10:1187. 10.3389/fimmu.2019.01187 31214171 PMC6555258

[95] Feske S. Calcium Signalling in Lymphocyte Activation and Disease. Nat Rev Immunol (2007) 7(9):690–702. 10.1038/nri2152 17703229

[96] Jeng MY Hull PA Fei M Kwon HS Tsou CL Kasler H. Metabolic Reprogramming of Human CD8(+) Memory T Cells Through Loss of SIRT1. J Exp Med (2018) 215(1):51–62. 10.1084/jem.20161066 29191913 PMC5748845

[97] Geltink RIK Kyle RL Pearce EL. Unraveling the Complex Interplay Between T Cell Metabolism and Function. Annu Rev Immunol (2018) 36:461–88. 10.1146/annurev-immunol-042617-053019 PMC6323527 29677474

[98] Chang HC Guarente L. SIRT1 and Other Sirtuins in Metabolism. Trends Endocrinol Metab (2014) 25(3):138–45. 10.1016/j.tem.2013.12.001S1043-2760(13)00206-3 PMC3943707 24388149

[99] Feng X Zhang L Acharya C An G Wen K Qiu L. Targeting CD38 Suppresses Induction and Function of T Regulatory Cells to Mitigate Immunosuppression in Multiple Myeloma. Clin Cancer Res (2017) 23(15):4290–300. 10.1158/1078-0432.CCR-16-3192 PMC5540790 28249894

[100] Newton R Priyadharshini B Turka LA. Immunometabolism of Regulatory T Cells. Nat Immunol (2016) 17(6):618–25. 10.1038/ni.3466 PMC5006394 27196520

[101] Huang L Xu H Peng G. TLR-Mediated Metabolic Reprogramming in the Tumor Microenvironment: Potential Novel Strategies for Cancer Immunotherapy. Cell Mol Immunol (2018) 15(5):428–37. 10.1038/cmi.2018.4 PMC6068099 29553135

[102] Morandi F Airoldi I Marimpietri D Bracci C Faini AC Gramignoli R. CD38, A Receptor With Multifunctional Activities: From Modulatory Functions on Regulatory Cell Subsets and Extracellular Vesicles, to a Target for Therapeutic Strategies. Cells (2019) 8(12):1527–44. 10.3390/cells8121527 PMC6953043 31783629

[103] Hartman WR Pelleymounter LL Moon I Kalari K Liu M Wu TY. CD38 Expression, Function, and Gene Resequencing in a Human Lymphoblastoid Cell Line-Based Model System. Leuk Lymphoma (2010) 51(7):1315–25. 10.3109/10428194.2010.483299 PMC2892000 20470215

[104] Glaria E Valledor AF. Roles of CD38 in the Immune Response to Infection. Cells (2020) 9(1):228–44. 10.3390/cells9010228 PMC7017097 31963337

[105] Chini EN Chini CCS Espindola Netto JM de Oliveira GC van Schooten W. The Pharmacology of CD38/NADase: An Emerging Target in Cancer and Diseases of Aging. Trends Pharmacol Sci (2018) 39(4):424–36. 10.1016/j.tips.2018.02.001 PMC5885288 29482842

[106] Samal B Sun Y Stearns G Xie C Suggs S McNiece I. Cloning and Characterization of the cDNA Encoding a Novel Human Pre-B-Cell Colony-Enhancing Factor. Mol Cell Biol (1994) 14(2):1431–7. 10.1128/mcb.14.2.1431-1437.1994 PMC358498 8289818

[107] Rongvaux A Shea RJ Mulks MH Gigot D Urbain J Leo O. Pre-B-Cell Colony-Enhancing Factor, Whose Expression Is Up-Regulated in Activated Lymphocytes, is a Nicotinamide Phosphoribosyltransferase, a Cytosolic Enzyme Involved in NAD Biosynthesis. Eur J Immunol (2002) 32(11):3225–34. 10.1002/1521-4141(200211)32:11<3225::AID-IMMU3225>3.0.CO;2-L 12555668

[108] Heske CM. Beyond Energy Metabolism: Exploiting the Additional Roles of NAMPT for Cancer Therapy. Front Oncol (2019) 9:1514. 10.3389/fonc.2019.01514 32010616 PMC6978772

[109] Hara N Yamada K Shibata T Osago H Tsuchiya M. Nicotinamide Phosphoribosyltransferase/Visfatin Does Not Catalyze Nicotinamide Mononucleotide Formation in Blood Plasma. PloS One (2011) 6(8):e22781. 10.1371/journal.pone.0022781 21826208 PMC3149623

[110] Galassi L Di Stefano M Brunetti L Orsomando G Amici A Ruggieri S. Characterization of Human Nicotinate Phosphoribosyltransferase: Kinetic Studies, Structure Prediction and Functional Analysis by Site-Directed Mutagenesis. Biochimie (2012) 94(2):300–9. 10.1016/j.biochi.2011.06.033 21742010

[111] Duarte-Pereira S Pereira-Castro I Silva SS Correia MG Neto C da Costa LT. Extensive Regulation of Nicotinate Phosphoribosyltransferase (NAPRT) Expression in Human Tissues and Tumors. Oncotarget (2016) 7(2):1973–83. 10.18632/oncotarget.6538 PMC4811510 26675378

[112] Manago A Audrito V Mazzola F Sorci L Gaudino F Gizzi K. Extracellular Nicotinate Phosphoribosyltransferase Binds Toll Like Receptor 4 and Mediates Inflammation. Nat Commun (2019) 10(1):4116. 10.1038/s41467-019-12055-2 31511522 PMC6739309

[113] Sayers SR Beavil RL Fine NHF Huang GC Choudhary P Pacholarz KJ. Structure-Functional Changes in eNAMPT at High Concentrations Mediate Mouse and Human Beta Cell Dysfunction in Type 2 Diabetes. Diabetologia (2020) 63(2):313–23. 10.1007/s00125-019-05029-y PMC6946736 31732790

[114] Revollo JR Korner A Mills KF Satoh A Wang T Garten A. Nampt/PBEF/Visfatin Regulates Insulin Secretion in Beta Cells as a Systemic NAD Biosynthetic Enzyme. Cell Metab (2007) 6(5):363–75. 10.1016/j.cmet.2007.09.003 PMC2098698 17983582

[115] Tanaka M Nozaki M Fukuhara A Segawa K Aoki N Matsuda M. Visfatin is Released From 3T3-L1 Adipocytes *via* a Non-Classical Pathway. Biochem Biophys Res Commun (2007) 359(2):194–201. 10.1016/j.bbrc.2007.05.096 17543285

[116] Grolla AA Torretta S Gnemmi I Amoruso A Orsomando G Gatti M. Nicotinamide Phosphoribosyltransferase (NAMPT/PBEF/visfatin) is a Tumoural Cytokine Released From Melanoma. Pigment Cell Melanoma Res (2015) 28(6):718–29. 10.1111/pcmr.12420 26358657

[117] Audrito V Manago A La Vecchia S Zamporlini F Vitale N Baroni G. Nicotinamide Phosphoribosyltransferase (NAMPT) as a Therapeutic Target in BRAF-Mutated Metastatic Melanoma. J Natl Cancer Inst (2018) 110(3):290–303. 10.1093/jnci/djx198 29309612

[118] Yoon MJ Yoshida M Johnson S Takikawa A Usui I Tobe K. SIRT1-Mediated eNAMPT Secretion From Adipose Tissue Regulates Hypothalamic NAD+ and Function in Mice. Cell Metab (2015) 21(5):706–17. 10.1016/j.cmet.2015.04.002 PMC4426056 25921090

[119] Sociali G Grozio A Caffa I Schuster S Becherini P Damonte P. SIRT6 Deacetylase Activity Regulates NAMPT Activity and NAD(P)(H) Pools in Cancer Cells. FASEB J (2019) 33(3):3704–17. 10.1096/fj.201800321R PMC6988859 30514106

[120] Yoshida M Satoh A Lin JB Mills KF Sasaki Y Rensing N. Extracellular Vesicle-Contained eNAMPT Delays Aging and Extends Lifespan in Mice. Cell Metab (2019) 30(2):329–42 e5. 10.1016/j.cmet.2019.05.015 31204283 PMC6687560

[121] Lu YB Chen CX Huang J Tian YX Xie X Yang P. Nicotinamide Phosphoribosyltransferase Secreted From Microglia *via* exosome during ischemic injury. J Neurochem (2019) 150(6):723–37. 10.1111/jnc.14811 31269239

[122] Chiarugi P Cirri P. Metabolic Exchanges Within Tumor Microenvironment. Cancer Lett (2015) 380(1):272–80. 10.1016/j.canlet.2015.10.027 26546872

[123] Kaymak I Williams KS Cantor JR Jones RG. Immunometabolic Interplay in the Tumor Microenvironment. Cancer Cell (2021) 39(1):28–37. 10.1016/j.ccell.2020.09.004 33125860 PMC7837268

[124] Camp SM Ceco E Evenoski CL Danilov SM Zhou T Chiang ET. Unique Toll-Like Receptor 4 Activation by NAMPT/PBEF Induces NFkappaB Signaling and Inflammatory Lung Injury. Sci Rep (2015) 5:13135. 10.1038/srep13135srep13135[pii 26272519 PMC4536637

[125] Torretta S Colombo G Travelli C Boumya S Lim D Genazzani AA. The Cytokine Nicotinamide Phosphoribosyltransferase (eNAMPT; PBEF; Visfatin) Acts as a Natural Antagonist of C-C Chemokine Receptor Type 5 (CCR5). Cells (2020) 9(2):495–509. 10.3390/cells9020496 PMC7072806 32098202

[126] Van den Bergh R Morin S Sass HJ Grzesiek S Vekemans M Florence E. Monocytes Contribute to Differential Immune Pressure on R5 Versus X4 HIV Through the Adipocytokine Visfatin/NAMPT. PloS One (2012) 7(4):e35074. 10.1371/journal.pone.0035074 22493731 PMC3320877

[127] Audrito V Serra S Brusa D Mazzola F Arruga F Vaisitti T. Extracellular Nicotinamide Phosphoribosyltransferase (NAMPT) Promotes M2 Macrophage Polarization in Chronic Lymphocytic Leukemia. Blood (2015) 125(1):111–23. 10.1182/blood-2014-07-589069blood-2014-07-589069[pii 25368373

[128] Travelli C Consonni FM Sangaletti S Storto M Morlacchi S Grolla AA. Nicotinamide Phosphoribosyltransferase (NAMPT) Acts as a Metabolic Gate for Mobilization of Myeloid-Derived Suppressor Cells. Cancer Res (2019) 79(8):1938–51. 10.1158/0008-5472.CAN-18-1544 30777853

[129] Audrito V Manago A Gaudino F Sorci L Messana VG Raffaelli N. NAD-Biosynthetic and Consuming Enzymes as Central Players of Metabolic Regulation of Innate and Adaptive Immune Responses in Cancer. Front Immunol (2019) 10:1720. 10.3389/fimmu.2019.01720 31402913 PMC6671870

[130] Naik J Themeli M de Jong-Korlaar R Ruiter RWJ Poddighe PJ Yuan H. CD38 as a Therapeutic Target for Adult Acute Myeloid Leukemia and T-Cell Acute Lymphoblastic Leukemia. Haematologica (2019) 104(3):e100–e3. 10.3324/haematol.2018.192757 PMC6395314 30190344

[131] Zeijlemaker W Grob T Meijer R Hanekamp D Kelder A Carbaat-Ham JC. CD34(+)CD38(-) Leukemic Stem Cell Frequency to Predict Outcome in Acute Myeloid Leukemia. Leukemia (2019) 33(5):1102–12. 10.1038/s41375-018-0326-3 30542144

[132] van de Donk N Richardson PG Malavasi F. CD38 Antibodies in Multiple Myeloma: Back to the Future. Blood (2018) 131(1):13–29. 10.1182/blood-2017-06-740944 29118010

[133] Damle RN Temburni S Calissano C Yancopoulos S Banapour T Sison C. CD38 Expression Labels an Activated Subset Within Chronic Lymphocytic Leukemia Clones Enriched in Proliferating B Cells. Blood (2007) 110(9):3352–9. 10.1182/blood-2007-04-083832 PMC2200908 17684154

[134] Malavasi F Deaglio S Damle R Cutrona G Ferrarini M Chiorazzi N. CD38 and Chronic Lymphocytic Leukemia: A Decade Later. Blood (2011) 118(13):3470–8. 10.1182/blood-2011-06-275610 PMC3574275 21765022

[135] Damle RN Wasil T Fais F Ghiotto F Valetto A Allen SL. Ig V Gene Mutation Status and CD38 Expression as Novel Prognostic Indicators in Chronic Lymphocytic Leukemia. Blood (1999) 94(6):1840–7. 10.1182/blood.V94.6.1840 10477712

[136] Durig J Naschar M Schmucker U Renzing-Kohler K Holter T Huttmann A. CD38 Expression Is an Important Prognostic Marker in Chronic Lymphocytic Leukaemia. Leukemia: Off J Leukemia Soc America Leukemia Res Fund UK (2002) 16(1):30–5. 10.1038/sj.leu.2402339 11840260

[137] Deaglio S Vaisitti T Zucchetto A Gattei V Malavasi F. CD38 as a Molecular Compass Guiding Topographical Decisions of Chronic Lymphocytic Leukemia Cells. Semin Cancer Biol (2010) 20(6):416–23. 10.1016/j.semcancer.2010.08.003 20817095

[138] Kumar SK Rajkumar SV Dispenzieri A Lacy MQ Hayman SR Buadi FK. Improved Survival in Multiple Myeloma and the Impact of Novel Therapies. Blood (2008) 111(5):2516–20. 10.1182/blood-2007-10-116129 PMC2254544 17975015

[139] Kawano Y Moschetta M Manier S Glavey S Gorgun GT Roccaro AM. Targeting the Bone Marrow Microenvironment in Multiple Myeloma. Immunol Rev (2015) 263(1):160–72. 10.1111/imr.12233 25510276

[140] Lonial S Weiss BM Usmani SZ Singhal S Chari A Bahlis NJ. Daratumumab Monotherapy in Patients With Treatment-Refractory Multiple Myeloma (SIRIUS): An Open-Label, Randomised, Phase 2 Trial. Lancet (2016) 387(10027):1551–60. 10.1016/S0140-6736(15)01120-4 26778538

[141] van der Veer MS de Weers M van Kessel B Bakker JM Wittebol S Parren PW. The Therapeutic Human CD38 Antibody Daratumumab Improves the Anti-Myeloma Effect of Newly Emerging Multi-Drug Therapies. Blood Cancer J (2011) 1(10):e41. 10.1038/bcj.2011.42 22829073 PMC3255255

[142] Khagi Y Mark TM. Potential Role of Daratumumab in the Treatment of Multiple Myeloma. Onco Targets Ther (2014) 7:1095–100. 10.2147/OTT.S49480 PMC4069139 24971019

[143] Garcia-Guerrero E Gotz R Doose S Sauer M Rodriguez-Gil A Nerreter T. Upregulation of CD38 Expression on Multiple Myeloma Cells by Novel HDAC6 Inhibitors Is a Class Effect and Augments the Efficacy of Daratumumab. Leukemia (2021) 35(1):201–14. 10.1038/s41375-020-0840-y PMC8318885 32350373

[144] Manna A Aulakh S Jani P Ahmed S Akhtar S Coignet M. Targeting CD38 Enhances the Antileukemic Activity of Ibrutinib in Chronic Lymphocytic Leukemia. Clin Cancer Res (2019) 25(13):3974–85. 10.1158/1078-0432.CCR-18-3412 PMC6744942 30940652

[145] Wo YJ Gan ASP Lim X Tay ISY Lim S Lim JCT. The Roles of CD38 and CD157 in the Solid Tumor Microenvironment and Cancer Immunotherapy. Cells (2019) 9(1):26–44. 10.3390/cells9010026 PMC7017359 31861847

[146] Antonioli L Yegutkin GG Pacher P Blandizzi C Hasko G. Anti-CD73 in Cancer Immunotherapy: Awakening New Opportunities. Trends Cancer (2016) 2(2):95–109. 10.1016/j.trecan.2016.01.003 27014745 PMC4800751

[147] Roh M Wainwright DA Wu JD Wan Y Zhang B. Targeting CD73 to Augment Cancer Immunotherapy. Curr Opin Pharmacol (2020) 53:66–76. 10.1016/j.coph.2020.07.001 32777746 PMC7669683

[148] Chen S Wainwright DA Wu JD Wan Y Matei DE Zhang Y. CD73: An Emerging Checkpoint for Cancer Immunotherapy. Immunotherapy (2019) 11(11):983–97. 10.2217/imt-2018-0200 PMC6609898 31223045

[149] Allard D Chrobak P Allard B Messaoudi N Stagg J. Targeting the CD73-Adenosine Axis in Immuno-Oncology. Immunol Lett (2019) 205:31–9. 10.1016/j.imlet.2018.05.001 29758241

[150] Perrot I Michaud HA Giraudon-Paoli M Augier S Docquier A Gros L. Blocking Antibodies Targeting the CD39/CD73 Immunosuppressive Pathway Unleash Immune Responses in Combination Cancer Therapies. Cell Rep (2019) 27(8):2411. 10.1016/j.celrep.2019.04.091 31116985

[151] Allard B Allard D Buisseret L Stagg J. The Adenosine Pathway in Immuno-Oncology. Nat Rev Clin Oncol (2020) 17(10):611–29. 10.1038/s41571-020-0382-2 32514148

[152] Hasmann M Schemainda I. FK866, a Highly Specific Noncompetitive Inhibitor of Nicotinamide Phosphoribosyltransferase, Represents a Novel Mechanism for Induction of Tumor Cell Apoptosis. Cancer Res (2003) 63(21):7436–42. 14612543

[153] Galli U Colombo G Travelli C Tron GC Genazzani AA Grolla AA. Recent Advances in NAMPT Inhibitors: A Novel Immunotherapic Strategy. Front Pharmacol (2020) 11:656. 10.3389/fphar.2020.00656 32477131 PMC7235340

[154] Dalamaga M Christodoulatos GS Mantzoros CS. The Role of Extracellular and Intracellular Nicotinamide Phosphoribosyl-Transferase in Cancer: Diagnostic and Therapeutic Perspectives and Challenges. Metabolism (2018) 82:72–87. 10.1016/j.metabol.2018.01.001 29330025

[155] Sampath D Zabka TS Misner DL O’Brien T Dragovich PS. Inhibition of Nicotinamide Phosphoribosyltransferase (NAMPT) as a Therapeutic Strategy in Cancer. Pharmacol Ther (2015) 151:16–31. 10.1016/j.pharmthera.2015.02.004 25709099

[156] Lucena-Cacace A Otero-Albiol D Jimenez-Garcia MP Peinado-Serrano J Carnero A. NAMPT Overexpression Induces Cancer Stemness and Defines a Novel Tumor Signature for Glioma Prognosis. Oncotarget (2017) 8(59):99514–30. 10.18632/oncotarget.20577 PMC5725111 29245920

[157] Zhu Y Liu J Park J Rai P Zhai RG. Subcellular Compartmentalization of NAD(+) and Its Role in Cancer: A sereNADe of Metabolic Melodies. Pharmacol Ther (2019) 200:27–41. 10.1016/j.pharmthera.2019.04.002 30974124 PMC7010080

[158] Chowdhry S Zanca C Rajkumar U Koga T Diao Y Raviram R. NAD Metabolic Dependency in Cancer is Shaped by Gene Amplification and Enhancer Remodelling. Nature (2019) 569(7757):570–5. 10.1038/s41586-019-1150-2 PMC7138021 31019297

[159] Tateishi K Wakimoto H Iafrate AJ Tanaka S Loebel F Lelic N. Extreme Vulnerability of IDH1 Mutant Cancers to NAD+ Depletion. Cancer Cell (2015) 28(6):773–84. 10.1016/j.ccell.2015.11.006 PMC4684594 26678339

[160] Travelli C Aprile S Mattoteia D Colombo G Clemente N Scanziani E. Identification of Potent Triazolylpyridine Nicotinamide Phosphoribosyltransferase (NAMPT) Inhibitors Bearing a 1,2,3-Triazole Tail Group. Eur J Med Chem (2019) 181:111576. 10.1016/j.ejmech.2019.111576 31400709

[161] Oita RC Camp SM Ma W Ceco E Harbeck M Singleton P. Novel Mechanism for Nicotinamide Phosphoribosyltransferase Inhibition of TNF-Alpha-Mediated Apoptosis in Human Lung Endothelial Cells. Am J Respir Cell Mol Biol (2018) 59(1):36–44. 10.1165/rcmb.2017-0155OC 29337590 PMC6039874

[162] Quijada H Bermudez T Kempf CL Valera DG Garcia AN Camp SM. Endothelial eNAMPT Amplifies Preclinical Acute Lung Injury: Efficacy of an eNAMPT-Neutralising mAb. Eur Respir J (2021) 57(5):2002536. 10.1183/13993003.02536-2020 33243842 PMC8100338

[163] Sun X Sun BL Babicheva A Vanderpool R Oita RC Casanova N. Direct Extracellular NAMPT Involvement in Pulmonary Hypertension and Vascular Remodeling. Transcriptional Regulation by SOX and HIF-2alpha. Am J Respir Cell Mol Biol (2020) 63(1):92–103. 10.1165/rcmb.2019-0164OC 32142369 PMC7328254

[164] Colombo G Clemente N Zito A Bracci C Colombo FS Sangaletti S. Neutralization of Extracellular NAMPT (Nicotinamide Phosphoribosyltransferase) Ameliorates Experimental Murine Colitis. J Mol Med (Berl) (2020) 98(4):595–612. 10.1007/s00109-020-01892-0 32338310

[165] Soncini D Caffa I Zoppoli G Cea M Cagnetta A Passalacqua M. Nicotinamide Phosphoribosyltransferase Promotes Epithelial-to-Mesenchymal Transition as a Soluble Factor Independent of Its Enzymatic Activity. J Biol Chem (2014) 289(49):34189–204. 10.1074/jbc.M114.594721 PMC4256351 25331943

